# What Percentage of Hairs Are Infected in Biopsies of Fungal Folliculitis?

**DOI:** 10.3390/dermatopathology10020020

**Published:** 2023-04-21

**Authors:** Sara Bourdages, Andrea Berger, Eric Hossler

**Affiliations:** 1 Geisinger Commonwealth School of Medicine, Scranton, PA 18510, USA; 2 Geisinger Department of Population Health Sciences, Danville, PA 17822, USA; 3 Departments of Dermatology and Pathology, Geisinger Medical Center, Danville, PA 17821, USA

**Keywords:** tinea capitis, Majocchi granuloma, fungal folliculitis, histopathology

## Abstract

Fungal folliculitis (including tinea capitis and Majocchi granuloma) has a wide range of clinical presentations, and biopsy may be obtained to distinguish this from other conditions with similar presentations. The study aims to evaluate the proportion of hairs infected in biopsies of fungal folliculitis. Copath records were searched for diagnoses of fungal folliculitis, tinea capitis and Majocchi granuloma between 1 January 2000 and 31 December 2020. Confirmed cases were pulled and reviewed by a dermatopathologist to count the total number of hairs on the sample and the total infected. Of 72 included cases, the median number of hair follicles per biopsy was 3 (IQR 1,4), and the median proportion of hairs infected was 54.2% (IQR 33.3%, 100.0%). Nineteen (26.4%) had only one hair included in the biopsy which was also an infected hair (100% of hairs were infected). The percentage of total hair follicles infected differed significantly depending upon location (*p* = 0.0443), with a smaller percentage of infected hairs in biopsies of tinea capitis. Clinicians should be cautious when using biopsy for diagnosis of fungal folliculitis, specifically, when there are few hairs in the specimen. Failure to capture infected hairs leads to false negative diagnoses.

## 1. Introduction

Dermatophytes are fungi that require keratin for growth. Fungal folliculitis is infection of a hair follicle caused by fungus and includes tinea capitis and Majocchi granuloma. These conditions have varying cutaneous presentations including erythematous papules, perifollicular or subcutaneous nodules, and abscesses; the presentation can vary based on the immune response of the host and the causative organism [1,2,3]. Biopsies may be obtained to distinguish fungal folliculitis from other conditions with similar cutaneous presentations. We have observed that not all hairs are affected in cases of fungal folliculitis, raising the possibility of false negative biopsy findings. Several studies have commented on microscopic characteristics of tinea capitis and Majocchi granuloma and the best staining techniques for identifying fungal elements on biopsy but do not address the percentage of hairs involved [4,5,6]. Our study aimed to determine the proportion of hairs infected on biopsies of fungal folliculitis.

## 2. Materials and Methods

After IRB approval (IRB number 2021-0144), the authors searched the electronic pathology database (Copath) for cases including the terms “fungal folliculitis”, “tinea capitis”, or “Majocchi granuloma” between 1 January 2000 and 31 December 2020. Pathology reports were reviewed, and 72 confirmed biopsies were included in the study. All specimens were examined by a single board-certified dermatopathologist who counted the total number of hairs in the sample and the number infected. Only follicles with a visible hair shaft were counted. For 4 mm punch biopsies, the specimen was already bisected and processed with six levels. Shave biopsies had already been submitted whole, bisected, trisected, or quadrisected based on size with 2 levels. All cuts of the specimen were examined, and hairs were tracked through the cuts in an effort to count each hair once. All specimens with a Periodic acid-Schiff (PAS) or Grocott’s methenamine silver (GMS) stain already completed were examined and correlated with the hematoxylin and eosin (H&E) stained specimen.

### Statistical Analysis

Patient and biopsy characteristics are summarized using means and standard deviations (S.D.) or medians and interquartile ranges (IQR) for continuous variables and frequency counts and percentages for categorical variables. The proportion of infected hair follicles was compared for categorical characteristics using Wilcoxon rank-sum or Kruskal–Wallis tests. Dwass-Steel-Critchlow-Fligner multiple comparison tests were used for pairwise comparisons of categorical variables with more than two levels. Statistical analysis was completed using SAS 9.4 (SAS Institute, Inc., Cary, NC, USA).

## 3. Results

There were 72 biopsies from 71 patients. The mean patient age was 54.0 years, and 60.6% of the patients were male. Patient demographics are displayed in Table 1. Forty-two (58.3%) of the biopsies came from an extremity, twelve (16.7%) were from the trunk, ten (13.9%) were from the scalp, seven (9.7%) were from the face or neck, and one (1.4%) was from an unspecified location. Nearly three quarters (73.6%) of the biopsies were performed using a punch technique and 26.4% were performed with a shave technique. Forty of the biopsies (55.6%) did not use stain, 40.3% used PAS, 2.8% used GMS, and 1.4% used both PAS and GMS. Fifteen biopsies (20.8%) had cultures taken. Sixty-one biopsies (84.7%) had a diagnosis of Majocchi granuloma and eleven (15.3%) had tinea capitis. See Table 2 for biopsy characteristics.

The median number of hair follicles included in a specimen was 3 (IQR 1,4) with a minimum of 1 and maximum of 54. The median number of infected hairs was 1 (IQR 1, 2) with a minimum of 1 and maximum of 49. The median proportion of hairs infected was 54.2% (IQR 33.3%, 100.0%) with a minimum of 11.1% and maximum of 100%. See Table 2. Nineteen biopsies (26.4%) had only one hair included in the biopsy which was also an infected hair (100% of hairs were infected). Table 3 describes the frequency of total number of hair follicles included by the number infected. 

The total number of hairs in the biopsy differed significantly based on location (*p* < 0.0001). Biopsies from the scalp had a greater median number of hairs compared to those from the trunk (median 9 versus 2 *p* = 0.0006). Biopsies from the scalp also had a greater median number of hairs compared to the extremities (median 9 versus 2, *p* = 0.0147). Table 4 describes the median total number of hairs on biopsy. 

The percentage of total hair follicles infected differed significantly depending on location (*p* = 0.0443), having a culture taken (0.0118), and diagnosis (0.0262). Biopsies taken from the extremities had a greater median proportion of infected hairs compared to those from the scalp (median 66.6% vs. 36.6%, *p* = 0.0344). Biopsies from patients with Majocchi granuloma had a greater median proportion of infected hairs compared to those with tinea capitis (median 66.6% vs. 50.0%, *p* = 0.0262). The proportion of infected hairs did not differ by sex, race, clinician specialty, technique, or stain used. See Table 5.

## 4. Discussion

Varying clinical presentations make histopathology a helpful tool for the diagnosis of fungal folliculitis. Although the histopathologic findings of fungal folliculitis are well described, there is limited information on the percentage of infected hairs in any given biopsy. In our study, we found that the medial proportion of infected hairs is 54.2%, with a wide variation in any given biopsy, ranging from 11.1 to 100%. Nineteen biopsies contained a single follicle, and of 15 biopsies that contained 2 hairs, 13 showed infection in only 1 hair. Additionally, a single infected follicle was found in biopsies containing up to 9 separate hair shafts. These findings illustrate that false negative biopsy results may be common, even in biopsies containing several hair shafts. This is important information for pathologists, as serial sections may be needed to reduce the chance of a false negative. This information is also important for clinicians when a biopsy fails to demonstrate fungal infections but clinical suspicion remains high. In this scenario, ancillary testing (potassium hydroxide scraping, trichoscopy, and/or culture) may be needed to definitively diagnose fungal infection. The biopsy is often viewed as the “gold standard” in the diagnosis of dermatologic conditions, but our study demonstrates that the possibility of a false negative biopsy must be considered given the substantial variation in follicle infection rate as well as the number of visualized hairs in any given biopsy. This caution is particularly warranted for scalp biopsies, where the rate of infected hairs was only 36.6% despite having the most hair follicles seen on biopsy.

Limitations of this study include the possibility of double counting hairs on biopsies with multiple hairs. Additionally, only 44.4% of the specimens in our study were stained with either GMS or PAS, which might have hindered the ability to detect hyphae in the specimens. We found routine H&E sections to be consistent with the stained counterparts for visualizing fungal elements in this study, however. Previous studies have shown an increase in the detection of hyphae with GMS or PAS staining compared to H&E staining [5,7]. Therefore, the percentage of infected hairs may be higher if routinely using stains. As a retrospective study, patient use of immune modulating or antifungal medications and chronic medical conditions were not controlled for but should be considered when examining biopsies for fungal folliculitis. 

Our study shows that 54% of hair follicles demonstrate fungal infection in confirmed cases of fungal infection. False negative biopsies remain a potential pitfall, particularly on the scalp. We propose examining multiple hairs, with routine serial sectioning and routine staining with PAS and/or GMS in cases where fungal folliculitis is suspected clinically. Additionally, pathologists may need to alert clinicians to the possibility of false negative results. Further studies are needed to determine the ideal approach to diagnosis of fungal folliculitis.

## Figures and Tables

**Table 1:** Patient Demographics.

	Patients *n* = 71
**Age, mean (S.D.)**	54.0 (20.5)
**Sex, n (%)**	
Female	28 (39.4%)
Male	43 (60.6%)
**Race**	
Black	1 (1.4%)
White	62 (87.3%)
Unknown	8 (11.3%)

**Table 2:** Biopsy Characteristics.

	Biopsies *n* = 72
**Location**	
Extremity	42 (58.3%)
Face/Neck	7 (9.7%)
Scalp	10 (13.9%)
Trunk	12 (16.7%)
Unspecified	1 (1.4%)
**Technique**	
Punch	53 (73.6%)
Shave	19 (26.4%)
**Stain**	
GMS	2 (2.8%)
PAS	29 (40.3%)
PAS/GMS	1 (1.4%)
None	40 (55.6%)
**Culture**	
Culture Taken	15 (20.8%)
No Culture Taken	57 (79.2%)
**Diagnosis**	
Majocchi granuloma	61 (84.7%)
Tinea capitis	11 (15.3%)
**Total Number of Hairs**	
Median (IQR)	3 (1, 4)
Minimum, Maximum	1, 54
**Total Number of Infected Hairs**	
Median (IQR)	1 (1, 2)
Minimum, Maximum	1, 49
**Percentage of Hairs Infected**	
Median (IQR)	54.2 (33.3, 100.0)
Minimum, Maximum	11.1, 100.0

**Table 3:** Frequency of Total Number of Hairs by Number of Hairs Infected.

Total Number of Hairs	Number of Hairs Infected	Percent Infected	*N*	%
1	1	100.0	19	26.4%
2	1	50.0	13	18.1%
2	2	100.0	2	2.8%
3	1	33.3	8	11.1%
3	2	66.6	3	4.2%
3	3	100.0	2	2.8%
4	1	25.0	5	6.9%
4	2	50.0	2	2.8%
4	3	75.0	2	2.8%
4	4	100.0	1	1.4%
5	1	20.0	1	1.4%
5	3	60.0	1	1.4%
5	4	80.0	1	1.4%
7	1	14.3	1	1.4%
8	1	12.5	1	1.4%
8	4	50.0	1	1.4%
8	7	87.5	1	1.4%
8	8	100.0	1	1.4%
9	1	11.1	1	1.4%
10	10	100.0	1	1.4%
12	6	50.0	1	1.4%
12	7	58.3	1	1.4%
13	3	23.1	1	1.4%
18	2	11.1	1	1.4%
54	49	90.7	1	1.4%

**Table 4:** Total Number of Hairs Based on Location.

			Total Number of Hairs		
	*n*	%	Median (IQR)	Minimum, Maximum	*p*-Value
**Location**					**<0.0001**
Extremity	42	58.3%	2 (1, 3)	1, 5	
Face/Neck	7	9.7%	8 (4, 10)	3, 12	
Scalp	10	13.9%	9 (5, 13)	2, 54	
Trunk	12	16.7%	2 (1, 4)	1, 5	

**Table 5:** Proportion of Hairs Infected by Biopsy Characteristics.

		Percentage of Total Hairs Infected		
	*N*	Median % (IQR)	Minimum %, Maximum %	*p*-Value
**Biopsies**	72			
**Sex**				0.3087
Female	29	75.0 (50.0, 100.0)	11.1, 100.0	
Male	43	50.0 (33.3, 100.0)	11.1, 100.0	
**Race**				1.0000
White	63	50.0 (33.3, 100.0)	11.1, 100.0	
Other/Unknown	9	66.6 (33.3, 100.0)	11.1, 100.0	
**Location**				0.0443
Extremity	42	66.6 (50.0, 100.0)	25.0, 100.0	
Face/Neck	7	50.0 (33.3, 87.5)	14.3, 100.0	
Scalp	10	36.6 (12.5, 50.0)	11.1, 100.0	
Trunk	12	62.5 (50.0, 100.0)	25.0, 100.0	
**Technique**				0.8902
Punch	53	58.3 (50.0, 100.0)	11.1, 100.0	
Shave	19	50.0 (33.3, 100.0)	23.1, 100.0	
**Stain**				0.0826
None	40	50.0 (33.3, 95.4)	11.1, 100.0	
GMS or PAS	32	90.0 (50.0, 100.0)	11.1, 100.0	
**Diagnosis**				0.0262
Majocchi granuloma	61	66.6 (50.0, 100.0)	14.3, 100.0	
Tinea capitis	11	50.0 (12.5, 87.5)	11.0, 100.0	

## Data Availability

The data presented in this study are contained within the article.
